# Development and in vitro testing of an orthodontic miniscrew for use in the mandible

**DOI:** 10.1007/s00056-024-00560-z

**Published:** 2024-11-26

**Authors:** Carolien A. J. Bauer, Pauline A. M. Karl, Juliana M. -K. Mielke, Christoph J. Roser, Christopher J. Lux, Mats Scheurer, Ludger Keilig, Christoph Bourauel, Lutz D. Hodecker

**Affiliations:** 1 Poliklinik für Kieferorthopädie, Universitätsklinikum Heidelberg, Im Neuenheimer Feld 400, 69120 Heidelberg, Germany; 2 Klinik und Poliklinik für Mund‑, Kiefer‑, Gesichtschirurgie, Universitätsklinikum Heidelberg, Im Neuenheimer Feld 400, 69120 Heidelberg, Germany; 3 Oralmedizinische Technologie, Zentrum für Zahn‑, Mund- und Kieferheilkunde, Universitätsklinikum Bonn, Welschnonnenstr. 17, 53111 Bonn, Germany

**Keywords:** Temporary anchorage devices, Anchorage, Primary stability, Fracture stability, Orthodontic mini-implant, Temporäre Verankerungselemente, Verankerung, Primärstabilität, Frakturstabilität, Kieferorthopädische Mini-Implantate

## Abstract

**Objective:**

Temporary anchorage devices (TADs) have been successfully used in the maxilla. However, in the mandible, lower success rates present a challenge in everyday clinical practice. A new TAD design will be presented that is intended to demonstrate optimization of the coupling structure as well as in the thread area for use in the mandible.

**Methods:**

Three TADs were examined: (A) Aarhus® system (68.99.33 A, Medicon, Tuttlingen, Germany), (B) BENEfit® orthodontic screw (ST-33-54209; PSM Medical, Gunningen, Germany) and (C) a new design with a two-part screw thread. The TADs were inserted into artificial bone blocks after predrilling to test primary stability. To test the fracture stability, the TADs were embedded in Technovit® 4004 (Heraeus Kulzer, Wehrheim, Germany) and torsional loaded at an angle of 90° until fracture. The threshold torque values occurring were recorded digitally. The statistical evaluation was carried out using the Kruskal–Wallis test with a post hoc test according to Bonferroni (*p* < 0.05).

**Results:**

The following values were measured for the insertion torque: A: 33.7 ± 3.3 Ncm; B: 57.1 ± 8.4 Ncm; C: 34.2 ± 1.4 Ncm. There were significant differences between A–B and B–C. The measured values for the fracture strength were as follows: A: 46.7 ± 3.5 Ncm; B: 64.2 ± 5.1 Ncm; C: 55.4 ± 5.1 Ncm. Significant differences were found between all groups.

**Conclusion:**

The adapted screw design has no negative influence on primary and fracture stability. Whether the design has a positive effect on the success rates in the mandible must be clarified in further clinical studies.

## Abstract (de)

**Zielsetzung:**

Temporäre Verankerungselemente (TADs) wurden erfolgreich im Oberkiefer eingesetzt. Die geringeren Erfolgsraten im Unterkiefer stellen jedoch eine Herausforderung in der klinischen Praxis dar. Es wird ein neues TAD-Design vorgestellt, das eine optimierte Kopplungsstruktur und einen optimierten Gewindebereich für den Einsatz im Unterkiefer aufweist.

**Methoden:**

Es wurden 3 TADs untersucht: (A) Aarhus® System (68.99.33 A; Medicon, Tuttlingen), (B) BENEfit® Orthodontic Screw (ST-33-54209; PSM Medical, Gunningen) und (C) ein neues Design mit einem 2‑teiligen Schraubengewinde. Die TADs wurden nach dem Vorbohren in künstliche Knochenblöcke eingesetzt, um die Primärstabilität zu testen. Zur Prüfung der Frakturstabilität wurden die TADs in Technovit® 4004 (Heraeus Kulzer, Wehrheim) eingebettet und bis zum Bruch in einem Winkel von 90° torsionsbelastet. Alle Torque-Schwellenwerte wurden digital aufgezeichnet. Die statistische Analyse erfolgte mittels Kruskal-Wallis-Test mit einem Post-hoc-Test nach Bonferroni (*p* < 0,05).

**Ergebnisse:**

Für das Eindrehmoment wurden folgende Werte gemessen: A: 33,7 ± 3,3 Ncm; B: 57,1 ± 8,4 Ncm; C: 34,2 ± 1,4 Ncm. Es gab signifikante Unterschiede zwischen A–B und B–C. Die gemessenen Werte für die Bruchfestigkeit waren wie folgt: A: 46,7 ± 3,5 Ncm; B: 64,2 ± 5,1 Ncm; C: 55,4 ± 5,1 Ncm. Es wurden signifikante Unterschiede zwischen allen Gruppen festgestellt.

**Schlussfolgerung:**

Das modifizierte Schraubendesign hat keinen negativen Einfluss auf die Primär- und die Frakturstabilität. Es sind weitere klinische Studien erforderlich, um festzustellen, ob sich das Design positiv auf die Erfolgsraten im Unterkiefer auswirkt.

## Introduction

The conventional approach to orthodontic tooth movement is based on the principle of Newton’s third law, which states that ‘action equals reaction’. Temporary anchorage devices (TAD) are well established in orthodontics to minimize unwanted tooth movement [9, 11]. TADs are mainly used in the maxilla. Reasons for screw loss include penetration into the mobile mucosa, poor oral hygiene and resulting mucosal inflammation, as well as failure to maintain the minimum distance to the tooth root [1, 22]. Due to the anatomical conditions, the use of TADs in the mandible is challenging in everyday clinical practice [28, 36, 44].

TADs typically have identical principal design, which can be divided into three sections. The upper part consists of the screw head and enables the connection with orthodontic elements. The middle section is the transgingival part, which is surrounded by the gingiva after insertion of the TAD. The length should be adapted to the gingival thickness. The lower threaded section is the part that is inserted into the bone. The thread pitch has an influence on the retention of the TAD. A tight thread can lead to higher stability and a small screw pitch can prevent lateral displacement of the TAD when an orthodontic force is applied [18, 35]. However, if the thread tightness is too high, the TAD may be more susceptible to fracture [40]. The screw tip and thread are usually self-drilling [47]. In general, self-drilling threads do not need to be predrilled and have a sharp-edged implant tip. Some manufacturers recommend predrilling before insertion not only for self-tapping threads, but also for self-drilling threads in areas with high bone density [47]. This refers to regions with thick cortical bone, such as it is present in the mandible. Predrilling in these areas reduces the torsional forces within the screw and the risk of fracture. It is recommended to use a predrill with a diameter 0.5 mm smaller than that of the mini-implant [8, 47]. The length and diameter of the TADs have a major influence on primary stability [38]. Short TADs should be used in areas with higher bone density, such as in the mandible, to increase stability and success rate [2]. The TADs available on the market vary in width, with most having a diameter of less than 3 mm. TADs with a diameter of 1.2 mm or more are ideal for the application of orthodontic forces [26].

**Fig. 1:**
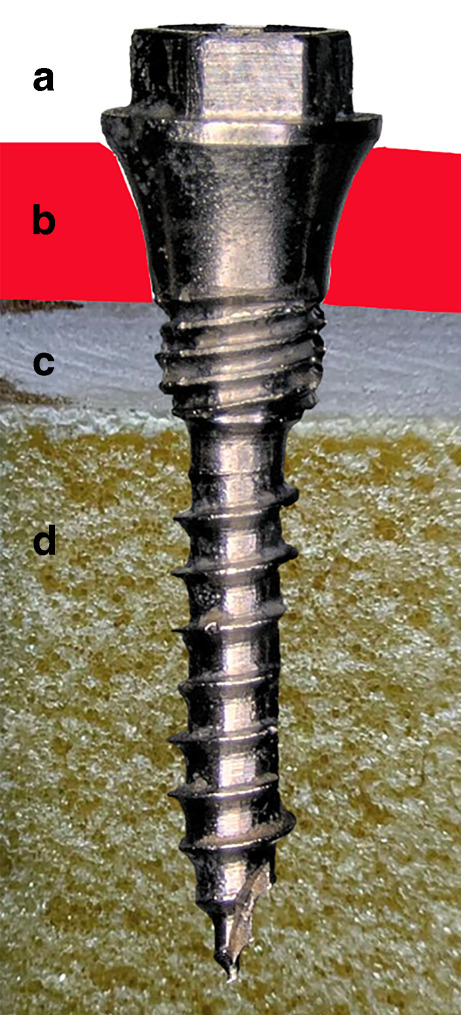
High-resolution image of the new screw design and schematic representation of the optimal positioning in the bone using an artificial bone block: a: connection area with internal thread, b: mucosa, c: cortical bone, d: cancellous bone Hochauflösende Aufnahme des neuen Schraubendesigns und schematische Darstellung der optimalen Positionierung im Knochen anhand eines künstlichen Knochenblocks: a: Verbindungsbereich mit Innengewinde, b: Schleimhaut, c: Kortikalis, d: Spongiosa

Before TADs are approved, they should be tested for their insertion torque and fracture stability. A high insertion torque is desirable as it increases primary stability, and a high fracture resistance is necessary to minimize the fracture risk of the screw [5]. The fracture torque or fracture stability should logically be significantly higher than the insertion torque. The limit values were defined in the Standard of the International Organization for Standardization ‘Dentistry—orthodontic anchor screws’ ISO 19023:2018 [10] which was adopted by European and German standard organizations (DIN EN ISO). The development of a screw designed to optimize the connection structure and thread specifically for the mandible is presented (Fig. 1). The screw design was developed based on current literature. In addition, the newly developed screw (MIRA screw) was analyzed for the properties required for approval. The null hypothesis of this study stated that the MIRA screw has no difference on insertion torque and fracture stability compared to two established TADs.

## Methods

**Fig. 2:**
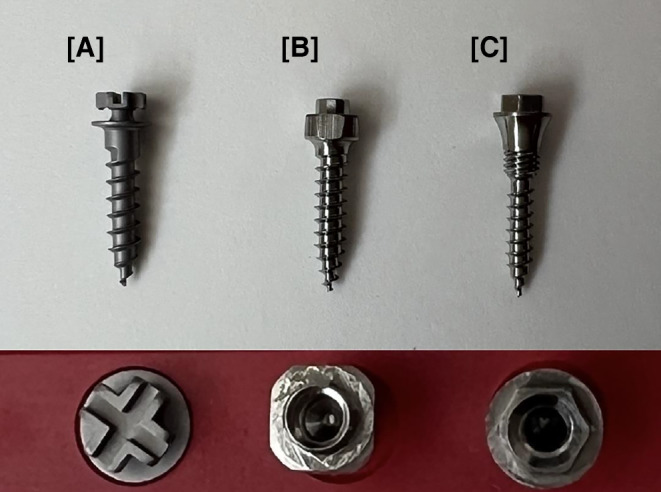
TADs used*. *From left to right: Aarhus® [A], BENEfit® [B] and the MIRA screw [C]. Frontal view of the screws and top view of the screw heads Verwendete TADs. Von links nach rechts: Aarhus® [A], BENEfit® [B] und die MIRA-Schraube [C]. Frontalansicht der Schrauben und Aufsicht auf die Schraubenköpfe

From a materials science perspective, two established TADs and the MIRA screw were analyzed in accordance with the DIN EN ISO 19023:2018 (Table 1; [10]). For each TAD, 10 screws were tested to check the dimensions and the insertion and fracture torque for insertion into artificial bone was recorded. Before the tests were carried out, the TADs were visually inspected for visible defects (Fig. 2). The manufacturing quality was examined, particularly with regard to the screw heads and threads.

**Table 1:** Temporary anchorage devices and manufacturers’ data Temporäre Verankerungselemente und Herstellerangaben

TAD	Manufacturer	Reference	Size (mm)	Thread diameter (mm)
Aarhus®	Medicon, Tuttlingen, Germany	68.99.33 A	2.0 × 8.4	Continuous 2.0
BENEfit®	PSM Medical GmbH, Tuttlingen, Germany	ST-33-54209	2.0 × 9.0	Continuous 2.0
MIRA	DEWIMED Medizintechnik GmbH, Tuttlingen, Germany	–	2.0 × 9.0	Two-part 1.6/2.0

### Verification of the dimensions

According to the DIN EN ISO 19023:2018, the screw dimensions must be checked using a measuring device with a maximum error limit of 0.05 mm [10]. In this study, this was done using an electronic caliper IP67 0–150 with a round depth gauge (TESA SA, Renens, Switzerland). The following dimensions were measured: overall length, head length, thread length, and diameter of the thread. As the MIRA screw is constructed with two different self-drilling threads, the diameter of the narrower and the wider thread were measured.

### Experimental setup

**Fig. 3:**
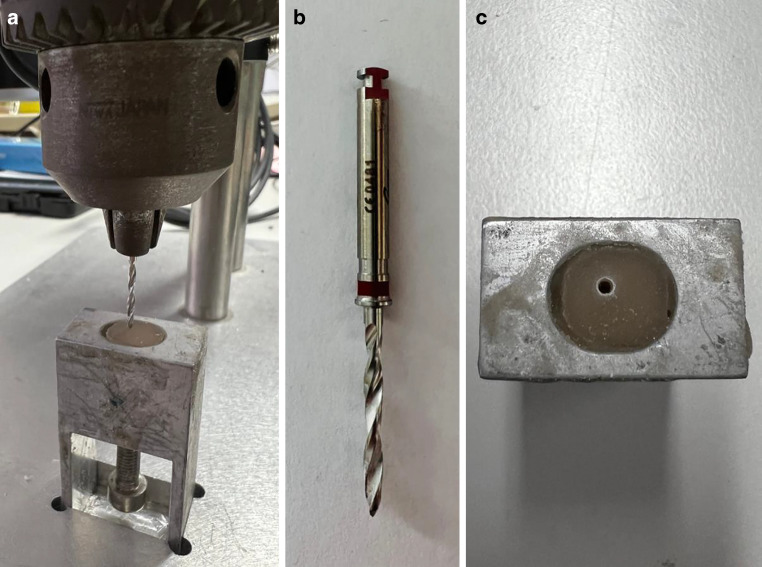
Measuring device with test holder and predrilled hole. **a** Measuring device with fixed predrill and test holder, with embedded artificial bone block. **b** Red predrill, diameter 1.4 mm, PSM Medical GmbH. **c** Finished predrilling in the embedded artificial bone block Messgerät mit Probenhalter und vorgebohrtem Loch. **a** Messgerät mit festem Vorbohrer und Probenhalter, mit eingebettetem künstlichem Knochenblock. **b** Roter Vorbohrer, Ø 1,4 mm, PSM Medical GmbH. **c** Fertige Vorbohrung im eingebetteten künstlichen Knochenblock

A measuring device complying to the ISO standard was used to determine the torque values [10, 37]. The measuring device consisted of a test specimen holder, a drilling device, and a torque measuring device (Fig. 3). During the test, the torques generated during screw insertion were measured in Ncm by the calibrated PCE-TM 80 torque meter (PCE Instruments, Meschede, Germany) and continuously recorded digitally using a self-developed software (LabVIEW, National Instruments, Munich, Germany). Artificial bone blocks (Ref 1522-319, Sawbones, Vashon Island, WA, USA), which were based on the anatomy of the mandible with compact and cancellous bone, were used to test the insertion torque. The upper 2 mm thick layer simulated the cortical bone and was filled with short epoxy resin fibers. The lower, thicker, and softer layer consisted of solid foam and simulated the cancellous bone. The bone blocks were additionally embedded in Technovit® 4000 (Heraeus Kulzer, Wehrheim, Germany) for secure and rotation-resistant fixation on the specimen holder. In order to check the fracture torque, the screw was embedded in the specimen holder using the polymer Technovit® 4004. The transgingival part and the head of the screw were left exposed.

### Performing the insertion torque test

**Fig. 4:**
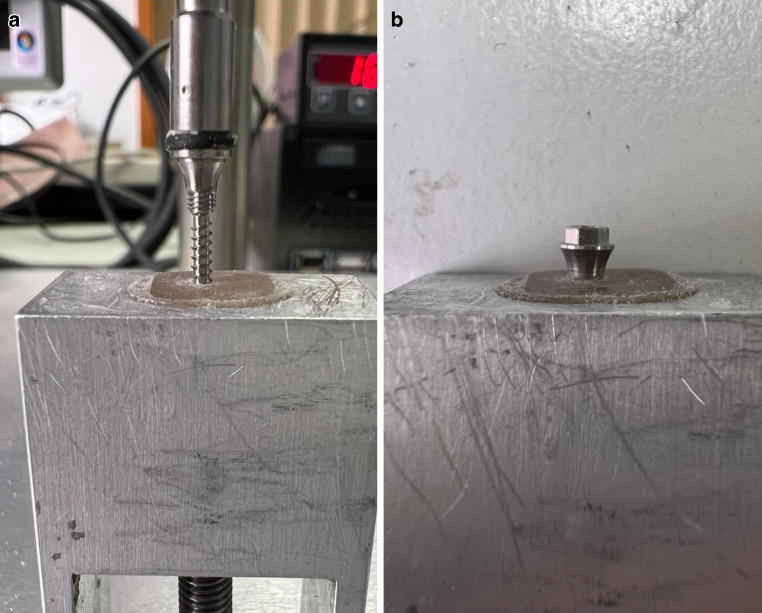
Testing of a temporary anchorage device (TAD) on primary stability*. ***a** TAD attachment with MIRA screw, which is attached to the measuring device and has already been inserted into the artificial bone block. **b** Completely inserted thread of the MIRA screw in the artificial bone block. No thread is visible from the outside Primärstabilitätsprüfung eines temporären Verankerungselements (TAD). **a** TAD-Aufsatz mit MIRA-Schraube, die am Messgerät befestigt ist und bereits in den künstlichen Knochenblock eingesetzt wurde. **b** Vollständig eingesetztes Gewinde der MIRA-Schraube im künstlichen Knochenblock. Von außen ist kein Gewinde sichtbar

First, a pilot hole was drilled vertical at a 90° angle to the artificial bone using a drill diameter of 1.4 mm (EASY DRIVER, PSM Medical, Gunningen, Germany) at five revolutions per minute (rpm) without cooling (Fig. 2). The pilot hole was drilled manually and only to a depth of 2 mm. To ensure even rotation, the starting point on the measuring device was marked with a pen and a timer was started so that a pilot hole could be drilled into the bone block at 5 rpm. The screws were then manually screwed into the predrilled hole at an insertion angle of 90° and 5 rpm without cooling until the final thread was no longer visible (Fig. 4). A total of 30 procedures were performed, of which 10 TADs per group were inserted into a new artificial bone block (*N* = 10).

### Performing the fracture torque test

**Fig. 5:**
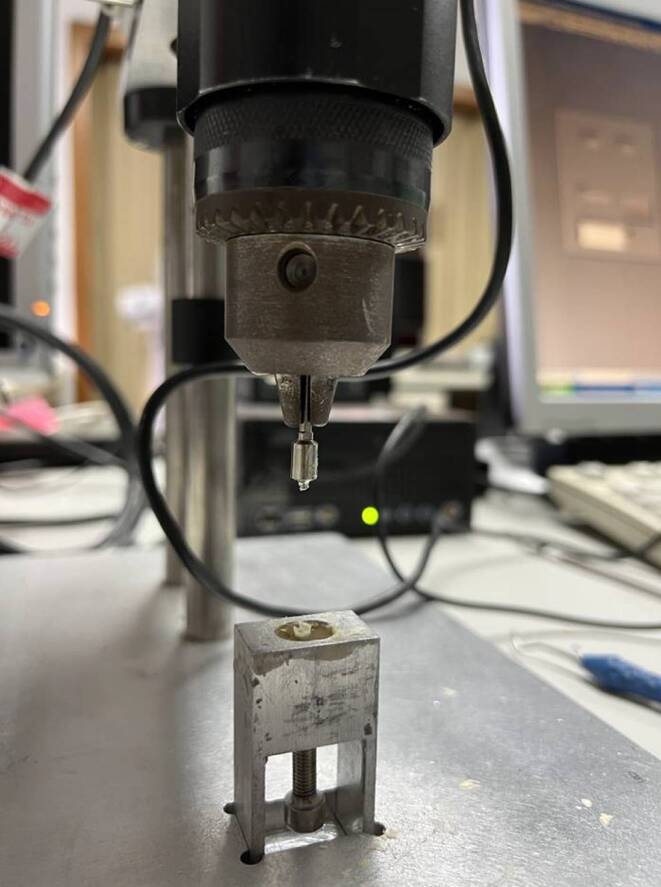
Testing of a temporary anchorage device (TAD) on fracture stability*. *Measuring device with a fractured BENEfit® screw. The test holder was filled with polymer, in which the TAD was embedded Bruchstabilitätstest eines temporären Verankerungselements (TAD). Messvorrichtung mit einer gebrochenen BENEfit®-Schraube. Der Probenhalter wurde mit Polymer gefüllt, in welches das TAD eingebettet wurde

As in the previous test, the starting point was marked on the measuring device with a pen and a timer was started. The TAD was turned manually at 5 rpm without cooling until fracture (Fig. 5). This was shown in the measurement curve by a sudden sharp drop. A total of 30 measurements were performed, of which 10 TADs per group (*N* = 10) were tested.

### Statistical evaluation

The experiments were statistically analyzed using the statistical software IBM SPSS version 28.0.1.0 for Windows (IBM, Armonk, NY, USA). The data from the verification of the dimensions were analyzed descriptively. The results of the tests for insertion torque and fracture torque were analyzed using the Kruskal–Wallis test with a post hoc test according to Bonferroni (*p* < 0.05).

## Results

**Fig. 6:**
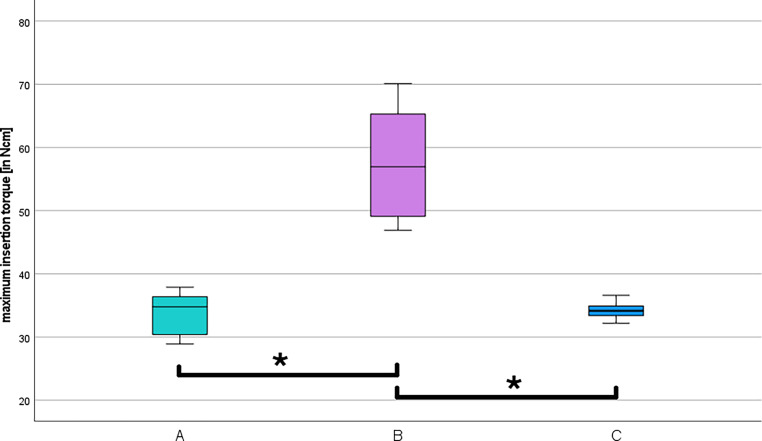
Evaluation of the maximum insertion torques*. *Illustration of the analyzed maximum insertion torques in Ncm for each temporary anchorage device (TAD) group examined in a box-and-whisker plot (A = Aarhus®, B = BENEfit®, C = MIRA screw). The asterisks illustrate the significant differences between the marked groups (*p* < 0.05) according to Kruskal–Wallis tests and post hoc Dunn–Bonferroni tests Auswertung der maximalen Eindrehmomente. Darstellung der analysierten maximalen Eindrehmomente in Ncm für jede untersuchte TAD(temporäres Verankerungselement)-Gruppe in einem Box-and-Whisker-Diagramm (A = Aarhus®, B = BENEfit®, C = MIRA-Schraube). Die Sternchen zeigen die signifikanten Unterschiede zwischen den markierten Gruppen (*p* < 0,05) gemäß Kruskal-Wallis-Test und Post-hoc-Dunn-Bonferroni-Test

Checking the dimensions provided the average measured values listed in Table 2. An average deviation of 0.03 mm for each dimension was measured. The analysis of the insertion torques for each TAD group showed that all measured TADs were within the standard (Fig. 6; [10]). The highest maximum insertion torque was measured in group B. This group also had the highest standard deviation (± 8.4 Ncm) with a mean value of 57.1 Ncm. Group C had the lowest standard deviation (± 1.4 Ncm) with a mean value of 34.2 Ncm. The mean maximum insertion torque for group A was 33.7 ± 3.3 Ncm. In Table 3 all values of the descriptive statistics are listed. The Kruskal–Wallis test was performed to analyze the differences in maximum insertion torque between the three groups. There were significant differences between groups A and B (*p* = 0.000) and B and C (*p* = 0.000). However, no significant differences were found between groups A and C (*p* = 0.939; Fig. 6).

**Table 2:** Descriptive statistics of the measured dimensions Deskriptive Statistik der gemessenen Dimensionen

		*N*	Minimum (mm)	Maximum (mm)	Mean value (mm)	Standard deviation (mm)
*BENEfit®*	Overall length	20	11.42	11.52	11.46	0.03
	Head length	20	2.40	2.50	2.43	0.03
	Thread length	20	8.99	9.08	9.03	0.03
	Diameter of the thread	20	1.98	2.03	2.01	0.01
*Aarhus®*	Overall length	20	11.59	11.62	11.61	0.01
	Head length	20	1.49	1.52	1.51	0.01
	Thread length	20	8.36	8.44	8.41	0.03
	Diameter of the thread	20	1.98	2.01	1.99	0.01
*MIRA*	Overall length	20	11.82	12.04	11.99	0.05
	Head length	20	1.12	1.21	1.18	0.02
	Thread length	20	8.96	9.03	9.01	0.02
	Upper diameter of the thread	20	1.96	2.16	2.12	0.04
	Lower diameter of the thread	20	1.50	1.66	1.62	0.03

**Table 3:** Descriptive statistics of the maximum insertion torques Deskriptive Statistik der maximalen Eindrehmomente

	Aarhus®	BENEfit®	MIRA
*N*	10	10	10
Mean value (Ncm)	33.7	57.1	34.2
Median (Ncm)	34.8	57	34.2
Standard deviation (Ncm)	3.3	8.4	1.4
Minimum (Ncm)	28.9	46.9	32.2
Maximum (Ncm)	37.9	70.1	36.6

**Fig. 7:**
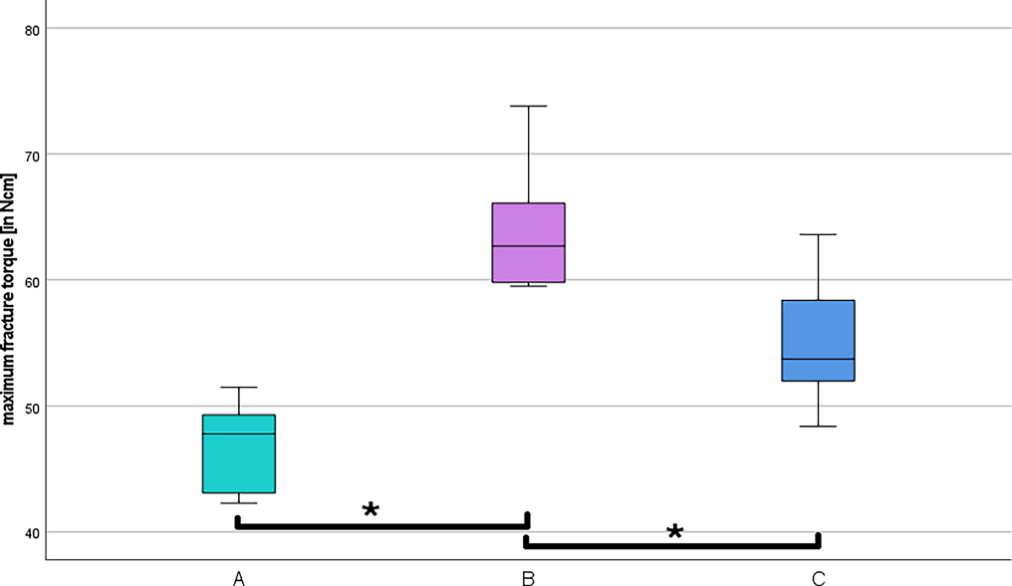
Evaluation of the maximum fracture torques*. *Illustration of the analyzed maximum fracture torques in Ncm for each tested screw group in a box-and-whisker plot (A = Aarhus®, B = BENEfit®, C = MIRA). The asterisks illustrate the significant differences between the marked groups (*p* < 0.05) according to Kruskal–Wallis tests and post hoc Dunn–Bonferroni tests Auswertung der maximalen Bruchdrehmomente. Darstellung der analysierten maximalen Bruchdrehmomente in Ncm für jede getestete Schraubengruppe in einem Box-and-Whisker-Diagramm (A = Aarhus®, B = BENEfit®, C = MIRA). Die Sternchen zeigen die signifikanten Unterschiede zwischen den markierten Gruppen (*p* < 0,05) gemäß Kruskal-Wallis-Test und Post-hoc-Dunn-Bonferroni-Test

The fracture torque tests for each group showed values that were also within the definitions of the standard (Fig. 7; [10]). The different fracture torques can be found in Table 4. The highest measurements were recorded in group B. In this group, the mean value and standard deviation were 64.2 ± 5.1 Ncm. The lowest fracture torques were found in group A. Here, the mean fracture torque was 46.7 ± 3.5 Ncm. In group C, the mean value and the standard deviation were 55.4 ± 5.1 Ncm. To check for significant differences, the Kruskal–Wallis test was performed again. Significant differences were found between groups A and B (*p* = 0.000) and A and C (*p* = 0.025). The differences between groups B and C were not significant (*p* = 0.126; Fig. 7).

**Table 4:** Descriptive statistics of the maximum breaking torques Deskriptive Statistik der maximalen Bruchdrehmomente

	Aarhus®	BENEfit®	MIRA
*N*	10	10	10
Mean value (Ncm)	46.7	64.2	55.4
Median (Ncm)	47.8	62.7	53.8
Standard deviation (Ncm)	3.5	5.1	5.1
Minimum (Ncm)	42.3	59.5	48.4
Maximum (Ncm)	51.5	73.8	63.6

**Fig. 8:**
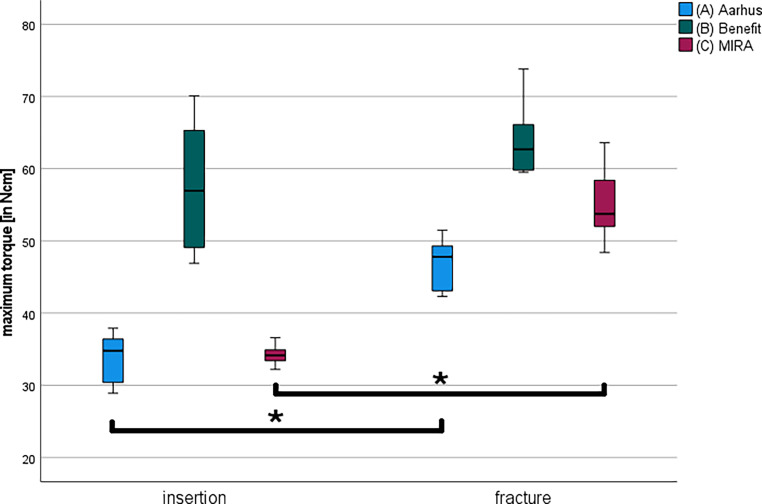
Evaluation of the maximum torques per temporary anchorage device (TAD) group. Illustration of the analyzed maximum insertion and fracture torques in Ncm for each tested TAD group in a box-and-whisker plot (A = Aarhus®, B = BENEfit®, C = MIRA). The asterisks indicate the significant differences between the marked groups (*p* < 0.05) according to the Mann–Whitney U‑test Auswertung der maximalen Drehmomente pro TAD(temporäres Verankerungselement)-Gruppe. Darstellung der analysierten maximalen Insertions- und Frakturdrehmomente in Ncm für jede getestete TAD-Gruppe in einem Box-and-Whisker-Diagramm (A = Aarhus®, B = BENEfit®, C = MIRA). Die Sternchen zeigen die signifikanten Unterschiede zwischen den markierten Gruppen (*p* < 0,05) gemäß dem Mann-Whitney-U-Test an

The Mann–Whitney U test for analyzing the insertion and fracture torques within a group showed significant differences for group A (*p* = < 0.001) and for group C (*p* = < 0.001). In contrast, the values for group B (*p* = 0.063) were not significant (Fig. 8).

## Discussion

The clinical success rates of interradicularly inserted TADs in the mandible were shown to be significantly lower compared to the maxilla [28]. TADs in the mandible are exposed to more masticatory forces, which could explain the increased fracture rate in the mandible [36]. The results of Toriya et al. suggest that bone density, the distance between the TADs and the neighboring roots and the vertical implantation angle are potential clinical signs of mandibular TAD failure [44]. The cortical bone is thicker, the keratinized gingiva is narrower, and the interradicular space is often small [43]. To develop a new screw design specifically for the mandible, the anatomical conditions, clinical requirements, and necessary material properties were considered. The material of the new TAD corresponds to the material of the established TADs (titanium-6-aluminium-4-vanadium, ISO 5832‑3 and ASTM F136).

The upper part of the new MIRA screw has a connection unit with an internal thread that can be used to fasten orthodontic appliances. The dimensions of the head are as small as possible (1 mm) to maximize patient comfort and enable suitable insertion for orthodontic appliances [42]. The centerpiece is tulip-shaped and has a smooth surface to avoid irritation of the mucosa. It has a length of 2 mm so that no thread is exposed even with a steeper insertion angle and varying gingival thickness. The lower part is 9 mm long and consists of two self-drilling threads. The lower thread is equipped with a self-drilling tip. The thread diameter is 1.6 mm and the thread pitch 0.7 mm. After insertion, this part is located in the interradicular area of the cancellous bone. Considering the interradicular bone volume in the mesiodistal direction and the thickness of the periodontal ligament of 0.2 mm, the ideal diameter of a TAD for this area ranges from 1.2 to 1.5 mm [34]. TADs for interradicular insertion should not be larger than 1.5 mm in diameter and 6 mm in length [29]. Animal studies have shown that not only direct contact but also a distance of less than 0.6 mm between the TAD and the root increases the risk of root resorption. Although thin TADs reduce the risk of root injury, they increase the risk of loss as the TAD may not be sufficiently stable [22]. For this reason, a lower thread diameter of 1.6 mm was chosen as a compromise. If the size of the diameter of the TADs is limited due to anatomical limitations, the length can be increased to improve primary stability [3]. However, the length should not exceed 9 mm [26]. The upper part of the lower section has a thread diameter of 2.0 mm and a thread pitch of 0.35 mm. The thread pitch is therefore half that of the lower part and serves to ensure that the upper part can anchor itself in the predrilled cortical bone. The larger thread diameter is intended to increase the insertion torque in the cortical bone and is not meant for placement in the cancellous bone [42]. Although a high insertion torque can increase primary stability, it can also have a negative effect on secondary stability, as excessive stresses can occur due to compression of the bone and soft tissue, microdamage and peri-implant bone resorption [8].

The TADs and their inserting instruments were visually inspected to identify any visible defects. This served as an initial assessment of the manufacturing quality. The TADs were measured using a digital measuring device [10]. However, it should be noted that precise three-dimensional measurement of the TADs is not possible with this method. In terms of functionality and biomechanical properties, this method is only of limited significance, which is why further tests were subsequently carried out.

Important criteria for the clinical performance of a new TAD are the torques that occur during insertion and fracture. For this reason, a material scientific investigation was performed in accordance with the DIN EN ISO 19023:2018 [10]. Two clinically proven TADs (Aarhus® [A], BENEfit® [B]), which have already been investigated in previous studies and are used clinically in the mandible [4, 27, 30, 31, 46] were used as comparison groups. It should be mentioned at this point that the BENEfit mini-implants were primarily developed for the maxilla. In this study, twice as many tests were carried out as required as a minimum (*N* = 10) [10].

When investigating the insertion torque, a pilot hole with a suitable diameter was drilled in advance. The size of the pilot hole should not exceed 1.65 mm for TADs with an outer diameter of 2 mm in order not to impair the primary stability [14]. Since the MIRA screw has an outer diameter of 2 mm in the upper thread but only 1.6 mm in the lower thread, a suitable pilot drill had to be selected. According to the literature, a pilot drill with a diameter of 1.4 mm was suitable for this purpose [17]. An artificial bone block (Sawbones) was used as the mandibular analogue [10, 16, 33]. The advantage of artificial bone blocks is that they are not subject to any aging process and always have the same thickness and density in the cortical and cancellous bone area. As the MIRA screw is intended for use in the interradicular region of the mandible, the thickness and density of the artificial cortical bone (2 mm) was in the medium range (0.86–3.03 mm) of the human mandible for this area [12, 20, 25, 33].

Polymer was used to embed the bone blocks with the TADs in the test holders [13, 47]. The polymer used to test the fracture torque had a bone-like modulus of elasticity of 2.3 GPa [6, 21, 32]. In addition, the homogeneous structure of the polymer enabled the tests to be carried out in a reproducible and comparable manner. The measuring device for recording the occurring torque values is an established method [10, 19]. By manually turning, the TADs were not inserted at the same speed. In clinical use, this can also occur during manual insertion. Thus, although the in vitro test procedure is similar to the clinical application, manual insertion is not exactly reproducible. However, manual insertion was demonstrated to result in the same primary stability as mechanical insertion [41].

The measurement of the insertion torque is of great clinical importance as it provides information about the primary stability. High primary stability is necessary to minimize the loss rate of the TADs [5, 45]. In agreement with the literature, the insertion torque was higher with the BENEfit® screw (B) than with the Aarhus® screw (A). This could be due to the fact that the two TAD groups have the same diameter of 2 mm but different lengths. A longer thread leads to higher torque during insertion [23, 39]. Although the TADs of group C (MIRA screws) and B had the same size, the insertion torque values of group C were closer to those of group A, with a lower standard deviation. This is probably due to the different geometry of the TADs [46]. The maximum insertion torque values of group C were between 32.2 and 36.6 Ncm and are comparable to the current study [7]. The results show that the MIRA screw can achieve sufficient primary stability despite the smaller lower screw diameter. The insertion torque values achieved are presumably due to the larger upper screw diameter with the fine thread for screwing into the cortical bone. TADs with a two-part thread in the cortical bone area appear to improve primary stability and mechanical retention [24].

The investigation of the fracture torque was intended to simulate the clinical situation of a fracture. However, this test setup can only be transferred to the clinical situation to a limited extent. The stable metal test holder forms a solid base. In this way, forces can be achieved that are not possible clinically. Increased torsional loads can lead to fractures at high forces [15]. Nevertheless, all measured values were in the range of 42.3 to 73.8 Ncm and are comparable with values from the literature [48]. In order to avoid necrosis of the bone, 40 Ncm should not be exceeded. As this is an in vitro study, in vivo studies should follow to investigate the real insertion torque. Each group differed significantly from the others. The fracture torque must be higher than the insertion torque [10]. If the values between the insertion and fracture torque are far apart, it can be assumed that the TADs can be inserted without fractures. This is particularly relevant in cases where the TAD is inserted into bone with a higher density than the artificial bone block used in this study. When comparing the torque values within the individual groups, it was noticeable that the MIRA and the Aarhus screw showed significant differences between the insertion and fracture torque. It can therefore be assumed that these TADs can be inserted safely. In contrast, the insertion and fracture moments of group B were not significantly different. This indicates a slightly increased risk of fracture during insertion.

The different TAD groups fractured at different locations. While those in group A fractured at two sites as expected (four TADs fractured within the cross slot, six TADs fractured at the implant head), only one predetermined breaking point occurred in the other groups [45]. In group B, as expected, all screws fractured below the screw head at the transgingival connection, while in group C the predetermined breaking point was at the junction between the two threads [15].

## Conclusions

- The insertion torque values of the MIRA screw were far below the fracture torque values, so that the adapted screw design seems to have no negative influence on primary and fracture stability.
- Whether the design has a positive effect on the clinical procedure and on the success rates of skeletal anchorage in the mandible must be clarified in clinical use and in further studies.

## Data Availability

All relevant data are available on request from the corresponding author.
